# Loss of genetic variability in social spiders: genetic and phylogenetic consequences of population subdivision and inbreeding

**DOI:** 10.1111/jeb.12022

**Published:** 2012-11-12

**Authors:** I Agnarsson, L Avilés, W P Maddison

**Affiliations:** * Department of Biology, University of VermontBurlington, VT, USA; † Institute of Biology, Scientific Research Centre of the Slovenian Academy of Sciences and ArtsLjubljana, Slovenia; ‡ Department of Biology, University of Puerto RicoSan Juan, PR, USA; § Department of Zoology, The University of British Columbia, University Blvd.Vancouver, BC, Canada; ¶ Department of Botany, The University of British Columbia, University Blvd.Vancouver, BC, Canada

**Keywords:** evolutionary dead end, heterosis, inbred social, inbreeding, phylogenetics

## Abstract

The consequences of population subdivision and inbreeding have been studied in many organisms, particularly in plants. However, most studies focus on the short-term consequences, such as inbreeding depression. To investigate the consequences of both population fragmentation and inbreeding for genetic variability in the longer term, we here make use of a natural inbreeding experiment in spiders, where sociality and accompanying population subdivision and inbreeding have evolved repeatedly. We use mitochondrial and nuclear data to infer phylogenetic relationships among 170 individuals of *Anelosimus* spiders representing 23 species. We then compare relative mitochondrial and nuclear genetic variability of the inbred social species and their outbred relatives. We focus on four independently derived social species and four subsocial species, including two outbred–inbred sister species pairs. We find that social species have 50% reduced mitochondrial sequence divergence. As inbreeding is not expected to reduce genetic variability in the maternally inherited mitochondrial genome, this suggests the loss of variation due to strong population subdivision, founder effects, small effective population sizes (colonies as individuals) and lineage turnover. Social species have < 10% of the nuclear genetic variability of the outbred species, also suggesting the loss of genetic variability through founder effects and/or inbreeding. Inbred sociality hence may result in reduction in variability through various processes. Sociality in most *Anelosimus* species probably arose relatively recently (0.1–2 mya), with even the oldest social lineages having failed to diversify. This is consistent with the hypothesis that inbred spider sociality represents an evolutionary dead end. Heterosis underlies a species potential to respond to environmental change and/or disease. Inbreeding and loss of genetic variability may thus limit diversification in social *Anelosimus* lineages and similarly pose a threat to many wild populations subject to habitat fragmentation or reduced population sizes.

## Introduction

There is a long-standing interest among biologists in the interplay, and consequences, of population subdivision and inbreeding (e.g. Baker, 1953, 1959; Vekemans *et al*., 1998; Schierup *et al*., 2000; Avilés & Purcell, 2012); especially well studied have been the potentially negative consequences of inbreeding in the short term, such as inbreeding depression, and the loss of genetic variability within populations thought to affect populations’ ability to adapt to local conditions and thus increase extinction risk (Reed *et al*., 2002; Woodworth *et al*., 2002). These consequences are a growing concern for conservation biology as human activities are fragmenting habitats, which results in decreased population sizes and increased inbreeding (Brook *et al*., 2002; Spielman *et al*., 2004b; Goulson *et al*., 2008; Van Rossum, 2008; Kang *et al*., 2008; Dixo *et al*., 2009; Schultz *et al*., 2009).

Most studies focus nearly exclusively on these short-term consequences of inbreeding, where the data primarily come from plants, frequently from laboratory experiments. However, the effects of population subdivision and inbreeding on the structure and amount of genetic variability may also have important long-term consequences, which are also crucially important, yet less well understood (Woodworth *et al*., 2002; Reed *et al*., 2003a; Reed & Frankham, 2003). The subdivision of a population into several subpopulations (demes) where mating is otherwise panmictic allows differentiation among demes, which can maintain genetic variability within the species as a whole (Charlesworth, 2003). Inbreeding, by reducing effective population sizes and the frequency of recombination events, should lead to the loss of genetic variability within demes (Wright *et al*., 2002; Charlesworth, 2003; Glemin *et al*., 2006; Mable, 2008). Thus, in typical outbred organisms, genetic variability is primarily explained by within-population differences, whereas in inbred species genetic variability is chiefly found between populations (Charlesworth, 2003). The effect of inbreeding on genetic variability at the species level is more difficult to predict. However, if deme isolation is sufficiently strong, inbreeding coupled with founder effects (such as colonizing events), selective sweeps and lineage turnover should purge genetic variability at the species level. In general, loss of variability due to these processes should be about double in the nuclear genome, compared to the mitochondrial; strongly inbred populations consists of homozygous genotypes resulting in shallower coalescences, and about half the time to common ancestry of alleles compared to a diploid population (Charlesworth, 2003). The study of Graustein *et al*. (2002), which is among the few to address the effect of mating system on genetic variability in an animal species with subdivided populations, broadly corroborates theoretical expectations (see Glemin *et al*., 2006, for an example in plants). These authors found that, compared to an outbred relative, the inbred *Caenorhabditis elegans* have at the species level an four- to six-fold reduction in nuclear diversity, whereas mitochondrial diversity was halved.

As pointed out by Charlesworth (2003) and Mable (2008), there is an urgent need for more studies, not the least in animals, that look at inbred species in nature, and the consequences of inbreeding and population subdivision in the context of related outbred species. The social spiders offer a natural inbreeding experiment. Within spiders, which are the largest known clade (over 43 000 known species, Platnick, 2012) of exclusively predatory animals, sociality has evolved about 20 times (Aviles, 1997; Agnarsson, 2006; Agnarsson *et al*., 2006, 2007; Johannesen *et al*., 2007; Lubin & Bilde, 2007; Avilés & Purcell, 2012), each time, with only one exception (Aviles, 1994), accompanied by a switch from outbreeding to inbreeding. This repeated evolution of a similar strategy is surprising given that the total number of social spider species is only slightly higher than the number of origins of sociality. This observation suggests that although inbred spider sociality may readily arise, it rarely persists long enough for speciation to take place within social lineages, or perhaps, that inbred sociality elevates extinction rates and thus slows diversification (Wickler & Seibt, 1993; Aviles, 1997; Agnarsson *et al*., 2006; Avilés & Purcell, 2012). Why might this be the case? Social spiders live in colonies made up of close relatives who mate exclusively, or nearly so, with one another to produce subsequent generations (Lubin & Crozier, 1985; Roeloffs & Riechert, 1988; Smith & Engel, 1994; Smith & Hagen, 1996; Johannesen *et al*., 2002; Bilde *et al*., 2005; Avilés & Bukowski, 2006; Johannesen *et al*., 2009). New colonies are established with relatively few individuals derived from a single-source colony, which should lower within-colony genetic variability. Founder events, population subdivision and inbreeding, combined with very limited or no intercolony migration and high turnover rates of colonies, should purge genetic variation at the level of metapopulations or species (Aviles, 1986, 1997; Smith & Hagen, 1996; Crouch & Lubin, 2001; Johannesen *et al*., 2002; Avilés & Purcell, 2012; Mattila *et al*., 2012; but see Johannesen *et al*., 2002, 2007). This may lower individual fitness and the ability of individuals and colonies to respond to natural selection exerted by environmental change, disease or parasitism.

Here, we use molecular data from one nuclear (28S rDNA) and three mitochondrial [cytochrome *c* oxidase 1 (COI), 16S rDNA and NADH dehydrogenase subunit 1 (ND1)] loci to estimate the effect of population subdivision and inbreeding on gene tree patterns and sequence divergence in inbred social species of the spider genus *Anelosimus* (Theridiidae). We do so across independent evolutionary replicas by comparing four independently evolved inbred social species with their outbred subsocial relatives. We also use these data to approximately estimate the age of the social lineages. We test two main hypotheses: (i) that inbred social species have shallower coalescences and lower sequence divergence than do their closest nonsocial relatives and (ii) the prediction of the dead-end hypothesis that sociality is confined to relatively recent lineages relative to the time of origin of subsociality.

## Materials and methods

We aimed to obtain 10–20 individuals of each focal *Anelosimus* species (Table 1), and sample multiple geographic localities for each species when possible. The samples of the social *Anelosimus eximius* and subsocial *A. baeza* and *A. studiosus* span a broad geographic range with at least 3000 km between the most distant localities. The Ecuadorian endemic *A. guacamayos* was also sampled across its known distribution. We were able to collect the remaining species only from a portion of their known distribution, limited to various localities in Ecuador. Of these species, the subsocial *A. elegans*, *A. tosum*, and the social *A. domingo*, are widespread in South America, whereas the social *A. oritoyacu* has only been collected in recent years from Ecuador, but is thought to occur also in Mexico (Agnarsson, 2006). In sum, two social and two subsocial species were sampled geographically broadly given their known ranges, and the remaining species are geographically undersampled. While we acknowledge that full geographic sampling of specimens would be optimal, we point out that (i) the undersampling is not biased towards social species, (ii) in a prior study we found high mitochondrial genetic diversity within small areas of Ecuador in the social *A. eximius* (Agnarsson *et al*., 2010) and (iii) undersampling should not affect the ratio between mitochondrial and nuclear genetic variability.

**Table 1:** Average pairwise, and maximum, sequence divergence of mitochondrial (Mit) and nuclear (Nuc) sequences within outbred subsocial (sub) and inbred social (soc) *Anelosimus* species. Comparisons are arranged in pairs of subsocial–social sister species (*elegans* vs *guacamayos*, *tosum* vs. *oritoyacu*) and comparing two subsocial (*baeza* and *studiosus*) to two social (*domingo* and *eximius*) species. Lower part of the table shows maximum sequence divergence between social and subsocial species pairs

	Mit pairwise	Mit max	Nuc pairwise	Nuc max
*elegans*	0.017	0.041	0.008	0.015
*guacamayos*	0.003	0.012	0.000	0.000
*tosum*	0.003	0.005	0.001	0.004
*oritoyacu*	0.000	0.001	0.000	0.000
*baeza*	0.015	0.033	0.002	0.008
*studiosus*	0.007	0.017	0.002	0.006
*domingo*	0.005	0.012	0.000	0.001
*eximius*	0.005	0.019	0.000	0.000
				
*guacamayos* and *elegans*		0.042		
*oritoyacu* and *tosum*		0.014		
*domingo* and *jabaquara*		0.023		

In addition, we use 1–7 individuals of other available *Anelosimus* species and the theridiines *Theridion* and *Coleosoma* as outgroups for phylogenetic analyses. Of the 55 known *Anelosimus* species, our analysis includes 24. The missing species mostly belong to three purely subsocial or solitary clades that are represented by at least two species each in our sample. The *eximius* group containing all but two of the social species is particularly important and is densely sampled with 14 of 18 known species included in the analysis. Taxonomic identity of individuals was based on morphological examination using recent taxonomic revisions (Agnarsson, 2005, 2006, 2012a, b; Agnarsson & Kuntner, 2005).

For each individual, sequences of the mitochondrial COI (1173 bp), 16S rDNA plus ND1 (577 bp) and the nuclear 28S rDNA (788 bp) were PCR-amplified. Additionally, the nuclear markers internal transcribed spacer region 2 (ITS2) and histone H3 were screened for variation relevant to our question here. Unfortunately, these were found to be invariable within the species here analysed (I. Agnarsson, unpublished) and thus not useful for our question. For extraction kits, primers and amplification conditions, see Agnarsson *et al*. (2007). PCR products were purified and sequenced by Macrogen Inc. (ABI 3730 sequencer) and proofread using the chromaseq module (Maddison & Maddison, 2011a) in mesquite 2.75 (Maddison & Maddison, 2011b), for details, see Agnarsson *et al*. (2007). Sequences were aligned using clustalx (Thompson *et al*., 1997) with gap opening/gap extension costs set to 24/6 following Maddison & Hedin (2003). For the nonprotein-coding genes, clustal alignments were subsequently subjected to minor editing using macclade (Maddison & Maddison, 2005). Manual editing was restricted to adjusting obviously misaligned regions mostly near the sequence ends. The GenBank accession numbers for the final sequences newly added here are: JX977169-JX977632.

Phylogenetic analyses were performed in TNT (Goloboff *et al*., 2008) and paup* (Swofford, 1999) using parsimony with a 1000 replicates using TBR branch swapping algorithm. Maximum-likelihood analyses were run in Garli (Zwickl, 2006), partitioning the analysis by loci and using the preferred model indicated by jModelTest (Posada, 2008) for each partition. Garli analyses were repeated 100 times until we could confirm that searches were converging on similar log likelihoods of the best tree and the tree maximizing the likelihood of the data among these replications was chosen. Under parsimony, all characters were equally weighted, excluding indels.

Uncorrected distance matrices of intraspecific sequence divergence, as well as the divergence between social and nonsocial sister species, were calculated using the dnadist package in phylip (Felsenstein, 2005), estimating both average and maximum sequence divergence. These measures were then compared between the four social species and the four most thoroughly sampled nonsocial species, including two social and nonsocial sister species comparisons.

Intra- and interspecific sequence divergence was used to infer lineage age, assuming approximate rates of mitochondrial evolution of 2–3% per million years, following Johannesen *et al*. (2007) for other social spiders. Additionally, an analysis of divergence time was conducted in beast and the associated software in that package as listed below (Drummond & Rambaut, 2007), under a strict molecular clock, as well as relaxed clock models. beast files were prepared in BEAUTI using the mitochondrial data set and with the default settings except defining Theridiinae and *Anelosimus* as monophyletic groups *a priori* and choosing either a strict or an exponential relaxed clock model. beast analyses were run for 10 000 000 generations and the first 10% discarded as burn-in, as determined by an analysis of the results in tracer. Results were summarized in tree annotator and visualized in figtree (Drummond & Rambaut, 2007). The main problem with such analyses is difficulty of calibration due to the lack of fossil record for *Anelosimus*. The only evidence is negative, that is, the complete absence of *Anelosimus* fossils in Baltic and Dominican amber. Some positive evidence comes from the sister lineage of Anelosiminae, the Theridiinae. Theridiine spiders are presently both common and abundant worldwide. Although the family Theridiidae is commonly found in Baltic amber, there are no confirmed members of the subfamily Theridiinae known from Baltic amber (about 40–50 mya) (Wunderlich, 2008); however, they do start appearing in young Dominican amber aged around 15–22 mya. Thus, the root of the tree here (Theridiinae plus *Anelosimus*) can be assumed to be at least 15–22 mya, but probably not much older than 40 mya. Thus, the one calibration point applied in beast was root age, where we tried the effects of ranging it from 15 to 40 mya, or setting the maximum age of *Anelosimus* to 15 mya.

To assess the degree of population subdivision, we calculated the fixation index (*F*_ST_) for the social species *A. eximius* for which we had much larger data sets available than for any other species. We use a data set of 16S rDNA and ND1 mitochondrial genes extracted from 170 specimens from 39 colonies from Ecuador and French Guiana (Agnarsson *et al*., 2010). *F*_ST_ was calculated using Arlequin (Excoffier *et al*., 2005). We calculated *F*_ST_ for both average and maximum sequence divergences; however, results did not differ.

## Results

**Fig. 1:**
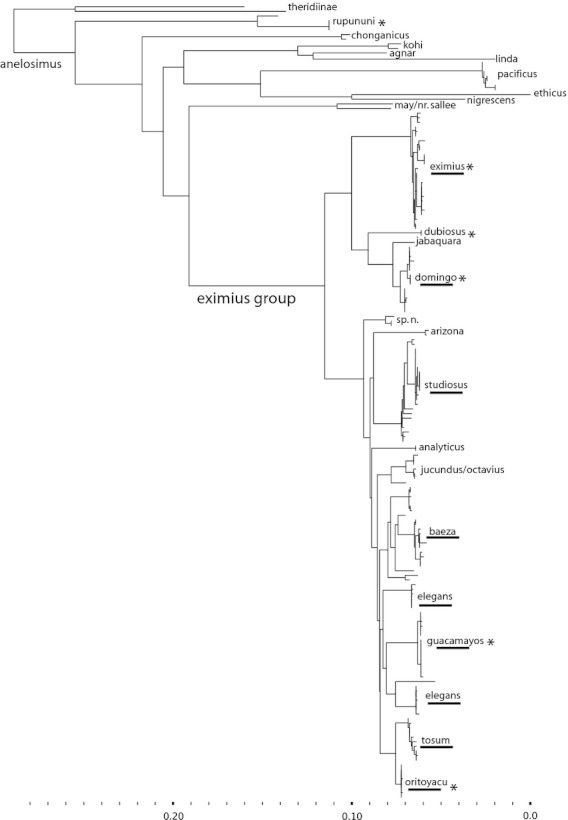
Results of the maximum-likelihood analysis with the lowest log likelihood (−4309.078, from Garli) of the mitochondrial data set. Branches are proportional to lengths; stars after species names indicate inbred social species; the eight focal species are underlined. The results broadly agree with Agnarsson *et al*.'s (#b^8^) species-level phylogeny and generally corroborate the morphology-based taxonomy of the group (Agnarsson, #b^2^). It is noteworthy that the social *Anelosimus guacamayos* nests within its putative sister species, the subsocial *Anelosimus elegans*. This may be due to incomplete lineage sorting or alternatively be an example of real species paraphyly. Sociality is expected to have arisen from a subsocial ancestral population – in this case, *A. guacamayos* may have evolved very recently from a population of *A. elegans*.

**Fig. 2:**
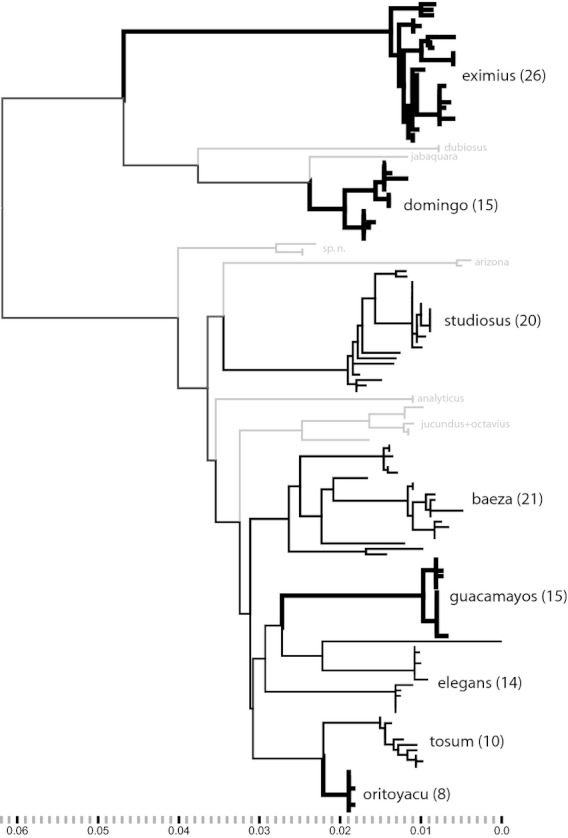
Results of maximum-likelihood analysis of the mitochondrial data for the focal species of the ‘*eximius* group’. Social species are indicated with bold lines; nonfocal taxa are deemphasized with grey. Numbers after species names indicate the number of individuals in gene tree. Note the relative shallowness of social lineages, particularly noticeable in the two sister pairs, the inbred social *Anelosimus guacamayos* vs. its outbred sister *Anelosimus elegans*, and the inbred social *Anelosimus oritoyacu* vs. its outbred sister *Anelosimus tosum*.

Unsurprisingly, the species-level phylogenies (Fig. 1 shows mitochondrial results based on maximum likelihood) agree well with our recent work (Agnarsson *et al*., 2007), confirming multiple origins of sociality (Fig. 2). The focal species were chosen to represent four independently evolved social species, and close relatives that we were able to obtain in relatively large samples – in two cases sister species of the social species (Fig. 2). On average, the inbred social species show shallower coalescences and less sequence divergence than do the outbred subsocial species (Fig. 4, Table 1). In the mitochondrial data, the social species have approximately half of the maximum sequence divergence of the subsocial species, on average (Table 1). The difference in genetic variability is particularly pronounced in the two cases where social species can be compared with subsocial sister species (by definition comparing lineages of equal ages, see Fig. 4, Table 1). The sequence divergence of the most divergent social species is *A. eximius* with about 0.5% average pairwise sequence divergence and 1.9% maximum intraspecific divergence. The other social species show average pairwise sequence divergences of 0.5% or less and maximum divergences of 1.2% (*A. domingo*, *A. guacamayos*) and 0.1% (*A. oritoyacu*) (Table 1). The subsocial species range from 0.2% (*A. tosum*) to 1.7% average pairwise sequence divergence and 0.5–4.1% maximum sequence divergence (Table 1). In the nuclear data, three of four social species showed no variability, whereas in the social *A. domingo*, maximum sequence divergence was 0.14%. The subsocial species all showed some 28S rDNA variability with maximum sequence divergence of 0.4% (*A. tosum*), 0.6% (*A. studiosus*), 0.8% (*A. baeza*) and 1.5% (*A. elegans*). The ratio of mitochondrial to nuclear genetic variability clearly differs between inbred social and outbred subsocial species (Fig. 4). Maximum sequence divergence between social–subsocial sister lineages was 4.2% between *A. guacamayos* and *A. elegans*, 1.4% between *A. oritoyacu* and *A. tosum* and 2.3% between *A. domingo* and the single available specimen of *A. jabaquara*.

**Fig. 3:**
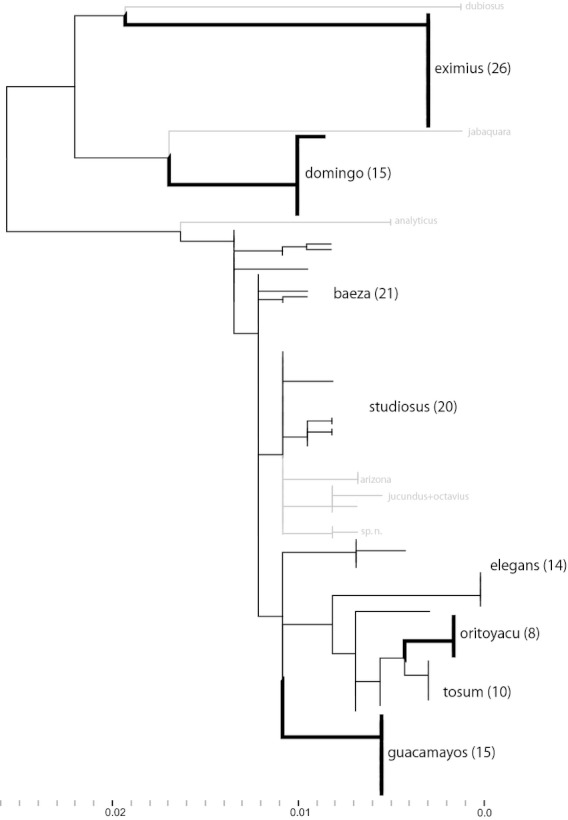
Results of maximum-likelihood analysis of the 28S rDNA nuclear data for the focal ‘*eximius* group’. Social species are indicated with bold lines; nonfocal taxa are deemphasized with grey. Numbers after species names indicate the number of individuals in gene tree. Note the complete lack of variation within social lineages other than *Anelosimus domingo* which shows a single base pair variation.

**Fig. 4:**
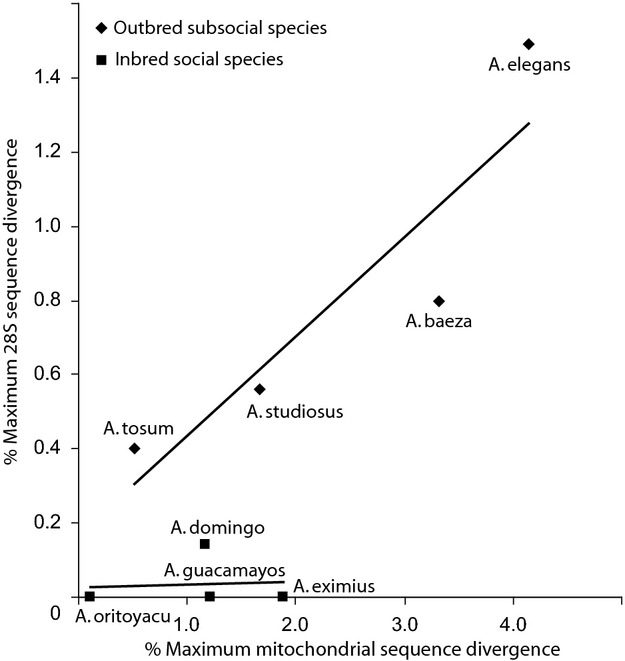
A comparison of maximum nuclear and mitochondrial sequence divergence of the four subsocial and the four inbred social species.

There is no clear evidence of geographic undersampling strongly confounding our findings. For example, the mitochondrial sequence divergence found in the subsocial *A. elegans* only sampled in a small part of its range within Ecuador was higher than that within the subsocial *A. studiosus* and *A. baeza* sampled much more geographically broadly. Similarly, the pairwise mitochondrial divergence was about the same in the social *A. domingo* sampled only within Ecuador and in the social *A. eximius* sampled also in the distant French Guinea. Also, geographic sampling of the sister species *A. elegans* (subsocial) and *A. guacamayos* (social) was about equal, with dramatically greater sequence divergence in the subsocial species.

Maximum mitochondrial interspecific sequence divergence within the genus *Anelosimus*, which is primitively subsocial, is approximately 26%. Assuming an approximate rate of mitochondrial evolution of 2–3% per million years following Johannesen *et al*. (2007), this implies that *Anelosimus*, and thus subsociality, originated a long time ago (perhaps 10–15 mya). This age agrees with the lack of fossil record of *Anelosimus* in Dominican amber, and with age estimates resulting from beast analysis, suggesting the age of *Anelosimus* ranging between 15 and 20 mya. In contrast, inbred sociality in all social species is relatively recent (Agnarsson *et al*., 2010). Sociality in *A. eximius* is difficult to bracket as its exact phylogenetic placement is uncertain (see Agnarsson *et al*., 2007; fig. 11), but minimal age of sociality in *A. eximius* based on the approximate rate of mitochondrial evolution is about 0.6–1 million years, or perhaps as much as 2 million years if accounting for halving the mitochondrial genetic variability (Table 2). In the other social species, inbred sociality presumably arose more recently. Based on maximum intraspecific sequence divergence (minimum age of sociality) and sequence divergence between social and subsocial sister pairs (maximum age of sociality), inbred sociality arose approximately 0.3–2 mya in *A. guacamayos*, 0.05–0.7 mya in *A. oritoyacu* and 0.4–1 mya in *A. domingo* (calculating divergences between specimens of *A. domingo* and the single available specimen of *A. jabaquara*, its putative sister species) (Table 2). beast analysis of divergence age using a maximum root age of 22–45 mya gives very similar estimates (Table 2), with inbred sociality arising approximately 1–2 mya in *A. eximius*, 0.4–0.8 mya in *A. guacamayos*, 0.2–0.4 mya in *A. oritoyacu* and 0.8–1.6 mya in *A. domingo* (Table 2). Analyses under a relaxed clock model with age of *Anelosimus* fixed at 15 mya give slightly higher age estimates for social species (Table 2). Although all these estimates thus indicate recent origins of sociality, we acknowledge that these estimates are imprecise due to the lack of calibration points and relatively few data. Further, all beast estimates have wide confidence intervals. Thus, further effort is needed to more tightly bracket the age of sociality in *Anelosimus* species.

**Table 2:** Estimates of the age of social lineages in *Anelosimus* using an estimated rate of COI evolution (2–3% rate), strict molecular clock analyses in beast based on root (Theridiinae plus Anelosiminae) age of 22 (S Clock 22) and 45 (S Clock 45) mya, and based on a relaxed clock analysis assuming the age of *Anelosimus* at 15 mya (R Clock)

Species	Age (2–3% rate)	Age (S Clock 22)	Age (S Clock 45)	Age (R Clock)
*eximius*	0.6–2 mya	1 mya	2 mya	2.5 mya
*guacamayos*	0.3–2 mya	0.4 mya	0.8 mya	1.2 mya
*domingo*	0.4–1 mya	0.8 mya	1.6 mya	1.7 mya
*oritoyacu*	0.05–0.7 mya	0.2 mya	0.4 mya	0.8 mya

For *A. eximius*, *F*_ST_ = 0.96; in other words, nearly all genetic variability at the species level is explained by the differences among colonies (populations), rather than the variation within them.

## Discussion

A switch to an inbred breeding system and strong population subdivision is expected to exert profound effects on the amount and distribution of genetic variability within and among populations (Graustein *et al*., 2002; Glemin *et al*., 2006; Granberg *et al*., 2008; Mattila *et al*., 2012). We have previously shown the effect of population subdivision and inbreeding on the repackaging of genetic variability among populations (Agnarsson *et al*., 2010), whereas our findings here demonstrate an effect on its quantity (see also Mattila *et al*., 2012). On average, the inbred social species show less sequence divergence compared with the outbred subsocial species. As sequence divergence roughly correlates with lineage age, it is unsurprising that not all four nonsocial species were more variable than all social species. For example, the nonsocial species with the lowest genetic variability, *A. tosum*, is less variable than the social *A. eximius*. However, the more appropriate comparison is with its sister species, the social *A. oritoyacu*: *A. tosum* is much more variable than *A. oritoyacu*.

As inbreeding is not, *per se*, expected to reduce genetic variability in the maternally inherited mitochondrial genome (Charlesworth & Wright, 2001), our findings suggest the loss of variation in the social species as a consequence of very strong population subdivision, coupled with founder effects and lineage extinction and replacement in time. Such extreme subdivision reduces effective population sizes. Lack of recombination between colonies retains this low variability, whereas lineage turnover can erode genetic variability at the metapopulation level (Aviles, 1997; Frankham, 1997; Woodworth *et al*., 2002; Gilligan *et al*., 2005). However, it may be that lineage turnover is rare and not a major force in limiting genetic variability. Instead, if the transition to sociality occurs within a single colony lineage, through depression of the dispersal phase and the establishment of sib-matings, social species may start out with nearly no variability due to founder effects. The shallowness of diversity within *A. oritoyacu* and *A. guacamayos*, which appear recent (Table 2), supports this scenario. Also congruent with this hypothesis is the placement of the social *A. guacamayos* within its putative sister species, the subsocial *A. elegans*. Although this may be due to incomplete lineage sorting, it is reasonable to view it as an example of real species paraphyly. Sociality is expected to have arisen from a subsocial ancestral population – in this case, *A. guacamayos* may have evolved recently from a population of *A. elegans*. This in turn hints that the relative depth of mitochondrial diversity within *A. eximius* and *A. domingo* is not a consequence of their retaining diversity through the transition to sociality, but rather a subsequent recovery of diversity afterwards as they persisted.

Although these conclusions are supported by our data, we note that further testing of these hypotheses is necessary through additional data. Our data have two main shortcomings: first that geographic sampling is far from complete so that true variability within species may be underestimated, and second, we are limited to mitochondrial data and a single nuclear marker. Our screening of additional nuclear markers (ITS2 and H3) suggests that these are not variable within species. As ITS2 is among the most variable nuclear markers commonly used in phylogenetics (e.g. Coleman, 2003; Agnarsson, 2010), other nuclear markers for which primers are available for spiders are unlikely to offer resolution to address the hypotheses presented here. Hence, additional standard PCR and phylogenetics are not likely to robustly test these hypotheses. Future work should instead focus on (i) more powerful genetic approaches, made available through next-generation sequencing technology, such as SNPs and phylogenomic tools (see e.g. Mattila *et al*., 2012 for an example involving social spiders), and (ii) a more thorough geographic sampling of specimens.

Given the observed uncorrected sequence divergence, the most divergent social species is *A. eximius* with about 2% intraspecific mtDNA divergence. The other social species show divergences of 1% or less, whereas the subsocial species range from 1% to 4%. Maximum mitochondrial sequence divergence within the genus *Anelosimus*, which is primitively subsocial, is 26%. Either assuming an approximate rate of mitochondrial evolution of 2–3% per million years (following Johannesen *et al*., 2007) or using a molecular clock analysis, this implies that whereas subsociality arose a long time ago, sociality in all social species is relatively recent. Sociality in *A. eximius* may be the oldest, up to 2 mya as estimated here, but is difficult to bracket due to the uncertain phylogenetic position of that species (see Agnarsson *et al*., 2007). Minimum age of sociality in *A. eximius* is as low as 0.6–1 mya. In the other social species, sociality appears to have arisen more recently (Table 2). The inbred social spiders have been hypothesized to represent an evolutionary dead end (Wickler & Seibt, 1993; Aviles, 1997), in part as a consequence of inbreeding (Agnarsson *et al*., 2006; Avilés & Purcell, 2012). The combination of relatively young age and low genetic variability observed here corroborates this hypothesis; inbred social lineages may rapidly go extinct (hence only young lineages are still extant) due to the inability to respond to environmental change and/or disease (Keller & Waller, 2002; Spielman *et al*., 2004a,b). Furthermore, even in lineages that have persisted long enough to have the opportunity to speciate, for example *A. eximius* which likely represents a relatively old social lineage (Table 2), there is little evidence for speciation within social lineages, again supporting the idea that inbred sociality restricts diversification. Johannesen *et al*. (2007) also noted that not all social spider lineages are very young. Sociality in some *Stegodyphus* species may have arisen as long as 3–4 mya. Thus, inbred spider sociality may not necessarily be short lived. However, that even really old social spider lineages have not speciated seems strong evidence that, even though inbred social lineages may persist for considerable periods, they tend not to speciate; hence, inbreeding restricts diversification.

A potential exception to lack of speciation of social lineages, given our current data, would arise if the clade containing *A. eximius* and *A. domingo* was primitively social, as it would be inferred under simple unordered parsimony. However, there are several lines of evidence to cast doubt on this scenario. First, *A. jabaquara*, the sister species of *A. domingo*, is not inbred social. Second, the placement of *A. eximius* in a clade with *A*. *domingo* is ambiguous. Agnarsson *et al*. (2007) found that different data sets disagreed on its position and that combined nuclear DNA, morphology and the mitochondrial ND1 all independently placed *A. eximius* outside that clade. Third, there may well be yet undiscovered subsocial species of this clade. *Anelosimus* has been especially well sampled in western South America (e.g. Agnarsson, 2006; Avilés *et al*., 2007), whereas eastern South America remains relatively poorly sampled. *Anelosimus eximius* and the ‘*domingo* clade’ (sensu Agnarsson, 2006) has all members either restricted to the east or overlapping the east; the *studiosus*/*jucundus* complex containing other species here discussed has all members either restricted to the west or overlapping the west. If there are as yet undiscovered species in the east, they are more likely to be subsocial as those species are more easily overlooked (small, often inconspicuous webs) than social ones.

In the nuclear data, the inbred social species have approximately 10% of the genetic variability of the subsocial species, suggesting that they are also loosing genetic variability through inbreeding (Reed *et al*., 2003b). Thus, our findings are very similar to those of Graustein *et al*. (2002). As for the mitochondrial data, these differences are especially notable in the two social–subsocial sister species comparisons that our sampling offers. The shift in mating system experienced by inbred sociality hence may result in reduction in variability through founder effects and extreme population subdivision and its various consequences including reduction in effective population sizes and inbreeding. These findings predict that habitat fragmentation that reduces population sizes, strongly isolates populations and leads to inbreeding may result in the loss of genetic variability and thus represent a serious concern for conservation biology (Brook *et al*., 2002; Keller & Waller, 2002; Reed *et al*., 2003c; Spielman *et al*., 2004b; Goulson *et al*., 2008; Kang *et al*., 2008; Van Rossum, 2008). This is especially so if the goals of conservation are to preserve not only standing biodiversity, but also the evolutionary potential of species and lineages over evolutionary time scales.

## Supporting information

**Appendix S1** Locality information for the specimens included in this study. Accession numbers are given for sequences from genbank, for accession numbers of taxa here added, see text.
